# The Effect of Alcohol on Near Point of Convergence and Accommodation

**DOI:** 10.22599/bioj.569

**Published:** 2026-11-09

**Authors:** Oseleonomhen Monica Odigie, Ese Francis Addeh, Clinton Ifeanyi Okechukwu, Ayishetu Oshoke Shuaibu

**Affiliations:** 1 University of Benin, NG; 2 St Mary Eye Clinic, Ago Iwoye, Ogun state, NG

**Keywords:** Alcohol consumption, Near point of convergence, Amplitude of Accommodation

## Abstract

**Background:**

The influence of alcohol on the central nervous system has been known to have an impact on different aspects of visual performance, a critical element for tasks like reading and driving. This study evaluated the effects of acute alcohol consumption on near visual components, namely, near point of convergence (NPC) and amplitude of accommodation (AA) in healthy individuals.

**Materials and Methods:**

This was a quasi-experimental pretest–posttest study conducted on 60 young participants (70% males and 30% females). The participants were divided into two groups: the alcohol group (mean age 24.1 ± 2.7 years) and the non-alcohol group (mean age 24.1 ± 2.8 years). NPC and AA were evaluated at baseline, 30 minutes, and 60 minutes in both groups. Independent *t*-tests and repeated-measures analysis of variance (ANOVA) were used to analyse the data; statistical significance was defined at *p* < 0.05.

**Results:**

NPC break and recovery values receded from 4.93 ± 3.76 cm to 7.53 ± 4.75 cm (*F* = 0.11, *p* = 0.745) and 6.37 ± 4.79 cm to 8.47 ± 4.69 cm (*F* = 0.01, *p* = 0.921), respectively, within one hour of ingestion of alcohol. Conversely, AA in the right and left eyes showed a significant decrease from 13.10 ± 2.89 D to 10.27 ± 3.38 D (*F* = 7.01, *p* = 0.013) and 12.13 ± 2.52 D to 10.00 ± 3.18 D (*F* = 7.01, *p* = 0.013), respectively.

**Conclusions:**

These results imply that accommodation is more susceptible to the acute effects of alcohol than convergence. When evaluating near-vision complaints, clinicians should take recent alcohol consumption into account. Alcohol-related near-vision impairment should be included in public health and traffic safety messaging.

## Introduction

Alcohol consumption is a major global health concern, contributing to mortality, disability, and impaired visual performance (Han *et al.*, 2022). Acute intoxication depresses central nervous system activity, leading to slowed eye movements, impaired accommodation, and disturbances in binocular vision (Karimi *et al.*, 2021; Martino *et al.*, 2021). Previous studies have demonstrated dose-dependent impairment in accommodation dynamics following alcohol consumption (Casares-López *et al.*, 2021a). These visual disruptions have significant functional implications for tasks such as reading and driving. In Nigeria, alcohol consumption is increasingly prevalent, with approximately one-third of adults engaging in hazardous drinking behaviours (Adeloye *et al.*, 2019). This is particularly concerning given the high burden of road traffic accidents, where alcohol use has been identified as a contributing factor (Ogazi and Edison, 2012). Together, these findings highlight the public health relevance of alcohol-induced visual impairment in both global and local contexts.

Despite evidence that alcohol degrades vision, near-point responses have been little studied. Specifically, few studies have measured the impact of acute, mild intoxication on the amplitude of accommodation (AA) or near point of convergence (NPC) in healthy individuals. According to a previous study (Munsamy *et al.*, 2016), participants displayed a slight but substantial retreating NPC and decreased fusional vergences at 0.10% BAC. However, these statistics have not been replicated in Nigerian or other African populations and are almost solely from high-income environments (such as South Africa). In West Africa, there are few peer-reviewed studies that specifically examine NPC or accommodation responses following alcohol consumption (Nkeremuzor *et al.*, 2025; Oboh *et al.*, 2020). There is, therefore, a glaring gap: in areas where drinking habits and biology may vary, acute, clinically significant alcohol doses and their immediate effects on the proximate triad are still poorly studied (Han *et al.*, 2022; Adeloye *et al.*, 2019). Although alcohol-related visual changes have been previously studied, there is little data in African populations utilising controlled quasi-experimental designs with matched control groups, which emphasises the study’s contextual importance.

This study attempts to close this gap by evaluating NPC and accommodative changes after controlled alcohol consumption in young adult Nigerians. Given that NPC and accommodation are routinely used in clinical assessment, their sensitivity to alcohol-induced changes may lead to misinterpretation if recent alcohol intake is not considered. Furthermore, impairments in convergence and accommodation may pose safety risks, particularly in activities such as driving, where precise visual function is required (Martino *et al.*, 2021). This study, therefore, contributes to both clinical understanding and public health awareness by demonstrating how acute alcohol consumption affects near visual function.

## Materials and Methods

### Study design

In order to assess the immediate effects of alcohol consumption on the NPC and AA in young adults in good health, this study used a quasi-experimental, pretest–posttest approach. Between August and September of 2025, all measurements were carried out at the University of Benin Optometry Teaching Clinic in Benin City, Nigeria. Convenience sampling was used to recruit healthy university students between the ages of 21 and 30 years.

Individuals with best-corrected visual acuity of 0.00 logMAR or better at 6 m and N5 at 40 cm, no history of binocular vision abnormalities, and no known ocular or systemic diseases that could impair accommodation or convergence were eligible to participate. People with a history of alcoholism, neurological conditions, ocular pathology, central nervous system medication usage, or uncorrected refractive problems were not included. The study did not include participants who were pregnant or who did not pass the alcohol use disorders identification test (AUDIT), a validated screening tool developed by the World Health Organization, to assess alcohol use behaviour. Participants scoring ≥8 (indicative of hazardous alcohol use) were excluded.

### Ethical approval

Ethical clearance (EC/UBEN/LSC.OPT/25/149) was obtained from the Department of Research and Ethics Committee of the Faculty of Optometry, University of Benin, Benin City, under the tenets of the Declaration of Helsinki. This ensured that all procedures performed on each subject were not against the public interest or inflicted unnecessary harm on them. Informed consent of all participants was obtained before any data was collected from them to ensure their full cooperation.

### Procedure

Participants were randomly assigned to either Group A (intervention group) or Group B (control group) using a computer-generated random number sequence. Participants in Group A (Intervention) received a measured dose of 250 ml of Toma red wine (12% alcohol by volume). Participants in Group B (Control) received an equal volume of 250 ml of a matched placebo beverage, specifically a dealcoholised red wine, which provided similar visual characteristics, taste, and calorie content without the ethanol component. All participants were instructed to consume their entire assigned beverage within a strict 10-minute time frame under the supervision of a research assistant who was not involved in data collection.

After a 30-minute absorption period, the Breath Alcohol Content (BrAC) was measured; those within the range of 0.8–1.0 g/L were assessed for NPC and AA. At the 60-minute absorption period, participants whose BrAC was measured and fell within the range of 0.7–1.1 g/L were also evaluated.

The testing was conducted in a standardised examination room with ambient lighting kept at 85 cd/m². To prevent circadian effects on accommodation and binocular function, all measurements were carried out during the day. All measurements were redone using the same techniques and equipment after alcohol consumption and the specified absorption period. The examiner was blinded to the group assignment of the participants.

### Assessment of NPC

A typical metre rule was used to determine the NPC. As the tip of a pen was gradually moved along the midline in the direction of the participants’ eyes, they were told to focus on it. The break point was measured in centimetres and was defined as the distance at which one eye deviated outward or diplopia was first detected. Additionally, the recovery point was recorded, where single vision was restored as the target was moved away. The mean of three trials was recorded for analysis after measurements were taken at baseline and again after drinking alcohol.

### Assessment of AA

The minus-lens-to-blur method with a near-accommodating target was used to measure AA. Minus lenses were added in front of the eye with the target until sustained blur was noted; participants were encouraged to maintain clear focus. The AA was calculated by adding the distance (in diopters) at which blur occurred with the last minus lens used. Measurements were taken monocularly in order to avoid the effects of convergence and replicated using a similar procedure after drinking alcohol.

### Data analysis

In this study, the data were analysed using SPSS version 22.0 (IBM Corp.). Baseline and post-consumption measures, as well as participant characteristics, were summarised using descriptive statistics. Alcohol and non-alcohol group values for the NPC and AA were compared using inferential statistics, such as independent *t*-tests. The threshold for statistical significance was fixed at *p* < 0.05. Within-group changes were evaluated using repeated-measures ANOVA at baseline, 30 minutes, and 60 minutes. The threshold for statistical significance was fixed at *p* < 0.05. To identify variations in measurements at various time intervals, post-hoc analyses using Bonferroni correction were carried out. The significance level was changed to α = 0.017 (0.05/3 comparisons).

## Results

A total of 60 participants were recruited and equally divided into alcohol (*n* = 30, mean age 24.1 ± 2.7 years) and non-alcohol group (*n* = 30, mean age 24.1 ± 2.8 years) groups. The demographic data and results of NPC and AA measurements for both the alcohol and non-alcohol groups across the different time intervals are presented in tables. Male participants (70%) constituted the majority in both groups (Table 1).

**Table 1:** Demographic data of alcohol and non-alcohol groups.


DEMOGRAPHIC DATA	ALCOHOL GROUP *n* (%)	NON-ALCOHOL GROUP *n* (%)

**Age (years)**		

21–25	20(66.7)	20(66.7)

26–30	10(33.3)	10(33.3)

**Gender**		

Male	21(70)	21(70)

Female	9(30)	9(30)


There was more recession in the NPCB and NPCR at baseline and at 30 minutes post-consumption interval in the alcohol group compared to the non-alcohol group. However, no statistically significant differences were observed between the two groups at all the time intervals (*p* > 0.05) (Table 2).

**Table 2:** Mean near point of convergence (cm) at baseline, 30, and 60 minutes.


VARIABLE	BASELINE		30 MINUTES		60 MINUTES	
		
	NPCB	NPCR	NPCB	NPCR	NPCB	NPCR

Alcohol Group	4.93 ± 3.76	6.37 ± 4.79	7.33 ± 4.13	8.47 ± 4.69	7.53 ± 4.75	8.33 ± 5.86

Non-Alcohol Group	3.97 ± 3.03	5.13 ± 3.79	6.00 ± 3.18	7.00 ± 3.78	8.33 ± 4.53	10.13 ± 4.99

*p* value	0.278	0.271	0.167	0.114	0.283	0.335


NPCB: near point of convergence break; NPCR: near point of convergence recovery.

There was a significant reduction in AOAR and AOAL in the alcohol group compared to the non-alcohol group at all-time intervals (*p* < 0.05) (Table 3).

**Table 3:** Mean amplitude of accommodation (D) at baseline, 30, and 60 minutes.


VARIABLE	BASELINE		30 MINUTES		60 MINUTES	
		
	AOAR	AOAL	AOAR	AOAL	AOAR	AOAL

Alcohol Group	13.10 ± 2.89	12.13 ± 2.52	11.83 ± 3.28	11.55 ± 3.46	10.27 ± 3.38	10.00 ± 3.18

Non-Alcohol	14.67 ± 2.43	14.93 ± 2.15	10.13 ± 2.57	9.60 ± 2.59	8.43 ± 3.38	8.13 ± 3.01

*p* values	0.027	0.000	0.029	0.016	0.040	0.023


AOAR: amplitude of accommodation right eye; AOAL: amplitude of accommodation left eye.

There was a recession of the NPCB from baseline (4.93 ± 3.76 cm) to 60 minutes (7.53 ± 4.75 cm) post-consumption interval in the alcohol group. There was also a recession in the NPCR from baseline (6.37 ± 4.79 cm) to 30 minutes (8.47 ± 4.69 cm) post-consumption interval in the alcohol group. No statistically significant differences were observed in the different time points for NPCB (*F* = 0.11, *p* = 0.745) and NPCR (*F* = 0.01, *p* = 0.921) in the alcohol group. There was a significant decrease in AOAR from baseline (13.10 ± 2.89 D) to 60 minutes (10.27 ± 3.38 D) post-consumption interval (F = 7.01, *p* = 0.013) in the alcohol group. In addition, a statistically significant difference in AOAR was observed between baseline and 60 minutes post-consumption interval (*t* = 3.49, *p* = 0.000). However, there was no statistically significant difference between baseline and 30 minutes post-consumption interval (*t* = 1.59, *p* = 0.118) and between 30 minutes and 60 minutes post-consumption interval (*t* = 1.82, *p* = 0 .074). There was also a significant decrease in AOAL from baseline (12.13 ± 2.52 D) to 60 minutes (10.00 ± 3.18 D) post-consumption (*F* = 7.01, *p* = 0.013) in the alcohol group. Furthermore, there was a statistical difference between baseline and 60 minutes post-consumption interval (*t* = 2.88, *p* = 0.006). No statistically significant differences were observed between baseline and 30 minutes (*t* = 0.75, *p* = 0.458) and between 30 minutes and 60 minutes (*t* = 1.81, *p* = 0.076) post-consumption intervals (Table 4).

**Table 4:** Summary of mean changes and within-group significance over time.


	GROUP	(MEAN ± SD)BASELINE	(MEAN ± SD)30 MINS	(MEAN ± SD)60 MINS	*P* VALUE

NPCB	Alcohol	4.93 ± 3.76	7.33 ± 4.13	7.53 ± 4.75	0.745

	Non-alcohol	3.97 ± 3.03	6.00 ± 3.18	8.33 ± 4.53	0.000

NPCR	Alcohol	6.37 ± 4.79	8.47 ± 4.69	8.33 ± 5.86	0.921

	Non-alcohol	5.13 ± 3.79	7.00 ± 3.78	10.13 ± 4.99	0.000

AOAR	Alcohol	13.10 ± 2.89	11.83 ± 3.28	10.27 ± 3.38	0.013

	Non-alcohol	14.67 ± 2.43	10.13 ± 2.57	8.43 ± 3.38	0.070

AOAL	Alcohol	12.13 ± 2.52	11.55 ± 3.46	10.00 ± 3.18	0.013

	Non-alcohol	14.93 ± 2.15	9.60 ± 2.59	8.13 ± 3.01	0.054


## Discussion

Our results demonstrate that near vision is negatively impacted by even moderate acute alcohol consumption. The mean NPC decreased numerically in those who consumed alcohol in our data. For instance, the NPC break (NPCB) receded from 4.93 ± 3.76 cm at baseline to 7.53 ± 4.75 cm at 60 minutes post-alcohol consumption, though this trend did not reach statistical significance (*F* = 0.11, *p* = 0.745). Furthermore, AA decreased significantly: the right eye’s amplitude decreased from 13.10 ± 2.89 D at baseline to 10.27 ± 3.38 D at 60 minutes (*F* = 7.01, *p* = 0.013), and the left eye’s amplitude decreased similarly. These alterations most likely resulted from ethanol’s depressive effects on muscle and neurological processes (Xie *et al.*, 2022).

In general, our findings agree with the published medical literature. Similar to our observed NPC trend, Munsamy *et al*. (2016) reported an average NPC shift of roughly 2.25 cm toward recession at moderate blood-alcohol levels; their study, like ours, found the convergence change to be slight. Similar to our noticeable decrease in AA, Casares-López *et al*. (2021a) found that wine consumption considerably retarded accommodative responses. Negative effects on accommodation and vergence with alcohol have also been reported by others (Miller *et al.*, 1985; Poças *et al.*, 2021). Even more severe deficits are seen in chronic heavy drinkers. Nagaraj *et al*. (2021) reported that alcoholics had significantly lower AA and greater exophoria at close distances than controls, suggesting a possible dose-response. Conversely, Hogan and Linfield (1983) reported very minor increases in static accommodation or minimal acute changes, but these findings have not been supported by more recent studies. Our findings support the more recent evidence that the near vision system is susceptible to the effects of alcohol (Casares-López *et al.*, 2021b; Martino *et al.*, 2021; Munsamy *et al.*, 2016).

The cerebral and muscle effects of alcohol provide a physiological explanation for these deficits. Ethanol reduces the neuronal drive to the extraocular and ciliary muscles by increasing inhibitory tone and decreasing cortical and subcortical neural activity (WHO, 2024; Xie *et al.*, 2022). When attempting near focus, the effect is a relaxation of the ciliary muscles (flatter lens) and relaxation of the vergence posture (eyes migrating outward). Alcohol’s myorelaxant action can reduce fusional convergence and slow accommodative response, which is consistent with animal and human models of sedation (Casares-López *et al.*, 2021b; Xie *et al.*, 2022). Our findings corroborate these theories and explain why inebriated viewers frequently perceive diplopia or reduced stereopsis. In addition, Xie *et al*. (2022) reported chronic alcohol use can lead to widespread deficits in the visual pathway.

There are important practical implications to these visual deficits. Convergence and accommodation are crucial for everyday tasks, including driving, reading, and scanning devices; however, these functions can be impaired even at legal-limit alcohol concentrations (~0.08% BAC). For example, driving necessitates precise depth estimation and quick concentration shifts (from the road to the dashboard); these tasks would be hindered by alcohol-compromised accommodation and convergence. Moderate alcohol consumption significantly reduces binocular vision and simulated driving skill (Martino *et al.*, 2021), indicating a connection between vision impairments and crash risk.

Since alcohol is implicated in roughly 25–30% of fatal collisions, the extra visual impairment is a serious issue (World Health Organization, 2024). Clinically, eye care professionals should consider recent alcohol consumption when patients show signs of new-onset convergence insufficiency or reduced near vision. Along with cognitive consequences, near-vision blurring and diplopia should be specifically mentioned as alcohol side effects in public health communications and alcohol warnings (Martino *et al.*, 2021; WHO, 2024). Emphasising that even mild intoxication can produce diplopia and depth-perception errors could improve awareness and safety compliance.

## Limitations

Our sample was small and made up of young, healthy volunteers; statistical power and generalisability may be limited. Higher dosages or longer-lasting effects cannot be addressed because we only examined one modest dose of alcohol and monitored performance for 60 minutes. The use of a single beverage variety in a laboratory setting might not accurately reflect the variability of drinking in the real world. Subtle asymmetries may have gone unnoticed since we did not capture precise eye movements or ocular dominance. Lastly, although comparable research (Martino *et al.*, 2021) predicts these might deteriorate under alcohol, we did not examine functional outcomes (e.g., driving errors or reading speed) in this study.

## Conclusion and Recommendations

As a result of ethanol’s CNS-depressant effect on the oculomotor system, acute alcohol ingestion dramatically reduces AA and causes NPC to recede. These visual abnormalities exacerbate the established hazards associated with alcohol intake. Educating the public and medical professionals on the effects of alcohol on vision is a crucial recommendation. On alcohol warning labels and in driver’s licensing programs, it could be required to draw attention to visual impairment as a risk of intoxication. Future studies should concentrate on larger and more diverse groups (such as elderly people with naturally lower accommodations), investigate various BAC levels and time periods, and use objective eye-tracking to assess vergence and accommodation mechanisms in the presence of alcohol.
